# Uremic Vascular Calcification: The Pathogenic Roles and Gastrointestinal Decontamination of Uremic Toxins

**DOI:** 10.3390/toxins12120812

**Published:** 2020-12-21

**Authors:** Chia-Ter Chao, Shih-Hua Lin

**Affiliations:** 1 Nephrology Division, Department of Medicine, National Taiwan University Hospital BeiHu Branch, Taipei 10845, Taiwan; b88401084@gmail.com; 2 Graduate Institute of Toxicology, National Taiwan University College of Medicine, Taipei 100233, Taiwan; 3 Nephrology Division, Department of Internal Medicine, National Taiwan University College of Medicine, Taipei 100233, Taiwan; 4 Department of Internal Medicine, Tri-Service General Hospital and National Defense Medical Center, Taipei 11490, Taiwan

**Keywords:** aortic calcification, chronic kidney disease, chronic kidney disease-mineral bone disorder, indoxyl sulfate, vascular calcification, vascular smooth muscle cell, oral adsorbent, uremic toxin

## Abstract

Uremic vascular calcification (VC) commonly occurs during advanced chronic kidney disease (CKD) and significantly increases cardiovascular morbidity and mortality. Uremic toxins are integral within VC pathogenesis, as they exhibit adverse vascular influences ranging from atherosclerosis, vascular inflammation, to VC. Experimental removal of these toxins, including small molecular (phosphate, trimethylamine-N-oxide), large molecular (fibroblast growth factor-23, cytokines), and protein-bound ones (indoxyl sulfate, *p*-cresyl sulfate), ameliorates VC. As most uremic toxins share a gut origin, interventions through gastrointestinal tract are expected to demonstrate particular efficacy. The “gastrointestinal decontamination” through the removal of toxin in situ or impediment of toxin absorption within the gastrointestinal tract is a practical and potential strategy to reduce uremic toxins. First and foremost, the modulation of gut microbiota through optimizing dietary composition, the use of prebiotics or probiotics, can be implemented. Other promising strategies such as reducing calcium load, minimizing intestinal phosphate absorption through the optimization of phosphate binders and the inhibition of gut luminal phosphate transporters, the administration of magnesium, and the use of oral toxin adsorbent for protein-bound uremic toxins may potentially counteract uremic VC. Novel agents such as tenapanor have been actively tested in clinical trials for their potential vascular benefits. Further advanced studies are still warranted to validate the beneficial effects of gastrointestinal decontamination in the retardation and treatment of uremic VC.

## 1. Introduction

Patients with impaired renal function, or chronic kidney disease (CKD), exhibit a high cardiovascular risk [1], which is frequently attributed to co-existing morbidities such as hypertension, diabetes, and dyslipidemia. Kidney-specific risk factors such as anemia, chronic inflammation, and mineral-bone disorders further compound the risk. Among the spectrum of cardiovascular diseases, vascular calcification (VC) is particularly dreadful because of its multifactorial pathophysiology and limited therapeutic options [2]. Studies from large CKD and end-stage renal disease (ESRD) cohorts report a high VC prevalence, up to 65% in the Chronic Renal Insufficiency Cohort (CRIC) [3] and 74% in the ESRD population [4]. The observed cardiovascular risk increases substantially during transition from non-dialysis CKD to ESRD, likely because of the progression of subclinical vascular lesions, especially VC. The inexorable path from VC to cardiovascular mortality has prompted researchers to identify useful diagnostic markers to predict the presence and course of VC, including protein-based [5], biochemistry-based [6], microRNA-based [7,8], and image-based ones [9].

Much has been learned from the pathophysiologic studies involving VC. Uremic toxins play a particularly critical role in the process of uremic VC, and targeted removal of these toxins may be an important approach for managing uremic VC. However, the existing literature fails to emphasize the multifaceted role of uremic toxin removal through the gastrointestinal route. In the following sections, we will provide an overview of the relationship between uremic toxins and VC; the plausible options for mitigating the adverse influences posed by uremic toxins; and, most importantly, the under-recognized role of gastrointestinal tract-based elimination of these toxins.

## 2. The Development and Propagation of Uremic VC

Generations of work have provided us with a deeper insight into how VC spawns and grows in the uremic environment. The prior theory of passive mineral deposition surrounding necrotic cores in the layers of the vascular wall has now been replaced by an active cell-mediated theory of osteoid matrix deposition, performed mainly by the phenotypically transformed vascular smooth muscle cells (VSMCs) [10]. A simplified anatomical classification divides VC into intimal and medical involvement, but there are frequent overlaps of the calcification process involving the two vascular layers. A multifaceted and integrated view of VC pathogenesis in patients with CKD seems to be a better explanation. These include osteoblastic trans-differentiation of VSMCs from metabolic noxious stimuli, divalent ion deregulation, the downregulation of endogenous calcification inhibitors, excessive levels of oxidative stress [2], chronic inflammation, and even the dysfunctional epigenetic machineries involving several vascular wall constituent cells [11]. Moreover, the precipitators of VC (e.g., uremia, diabetes mellitus, and vascular ageing) and the resultant VC are dually responsible for the propagation and the adverse consequences.

## 3. Uremic Toxins as a Significant Contributor to VC

Among the pathogenesis of VC, uremic toxins expectedly assume importance over time as CKD progresses. A majority of the uremic toxins exhibit adverse vascular effects, ranging from atherosclerosis, vascular inflammation, to VC. Clinical studies consistently show that levels of multiple uremic toxins closely correlate with the severity of uremic VC. Large cohort studies have shown that higher serum phosphate levels are independently associated with an increased mortality and accelerated progression of VC among CKD patients [12], while an elevated serum uric acid level also predicts worse survival among those with ESRD [13]. Serum levels of other uremic solutes such as trimethylamine-N-oxide (TMAO) have been reported to be surrogate markers of atherosclerotic burden among CKD patients [14]. Higher serum protein-bound uremic toxins such as indoxyl sulfate (IS) are also associated with a greater prevalence of aortic calcification and vascular stiffness among CKD patients [15] and paralleled the severity of VC among patients with ESRD [16]. It is then reasonable to infer that uremic toxins, besides as a surrogate for the degree of renal function decline, can directly contribute to the emergence of uremic VC.

## 4. Classification of Uremic Toxins

As proposed by the European Uremic Toxin (EUTox) workgroup [17], candidate uremic toxins are recognized when they are retained during periods of renal function decline. An increasing proportion of these compounds is revealed to exhibit harmful health influences. These uremic toxins can be simply divided according to their molecular size, water solubility, and protein-binding status. We will provide a brief outline of some of the well-established VC-relevant uremic toxins below.

### 4.1. Small Molecular Eeight Less Than 500 Da

These are prototypic, including urea, creatinine, calcium, inorganic phosphate, and polyamines [17], although currently, some researchers have excluded calcium and phosphate from this list. Classic uremic toxins such as urea, once thought to be health-neutral, and have been found to induce alterations in intestinal barrier, facilitating bacterial translocations and endothelial dysfunction [18]. The list of small molecular uremic toxins now evolves to include uric acid and acrolein because of their close relationship with oxidative metabolism of endogenous proteins and lipids. Trimethylamine (TMA) and TMAO have also been shown to increase stepwise with deteriorating renal function, and the administration of these molecules to experimental animals can induce renal damages, vascular inflammation, and alter cholesterol metabolism [19].

#### Small Molecular Uremic Toxins and Uremic VC

Experimentally, a myriad of evidence indicates that the retention of small molecular uremic toxins, such as inorganic phosphate, precipitates VC. The model of phosphate-induced osteoblastic trans-differentiation of VSMCs has assumed widespread popularity as an experimental approach for studying VC [2]. Mechanisms underlying the phenotypic switch include not only the upregulation of Wnt/β-catenin, bone morphogenetic protein (BMP), and pro-inflammatory nuclear factor-κB (NF-κB) signaling, as well as the suppression of Klotho expression, but also potentially epigenetic anti-calcification machineries [20,21]. Inorganic phosphate was also shown to retard the process of osteoclastogenesis through downregulating miR-223 and potentially de-inhibiting the osteoblastic activity in uremic blood vessels [22]. TMAO, another small uremic solute, is also shown to promote vascular inflammation and VC through activating inflammasomes and inducing NF-κB expressions [23]. Exposure to monosodium urates and increased uric acid concentrations, in an in vitro model, also induces an upregulation of Wnt3a and RUNX2 expressions in cultured VSMCs [24].

### 4.2. Large Molecular (Middle Molecule) Uremic Toxins 

These large molecules are more difficult to tackle compared with small molecular ones, because of the inefficient removal using conventional dialysis modalities. The terminology of these molecules can be diverse, ranging from middle molecules [25], large molecules [26], to large middle molecules [27], but the members of this category of uremic toxins are essentially similar. These toxins, including fibroblast growth factor-23 (FGF-23); leptin; interleukin families such as interleukin-1β (IL-1β), IL-6, and IL-18; tumor necrosis factor-α (TNF-α); and advanced glycation endproducts (AGEs), also play an important link between CKD and cardiovascular morbidity [26].

#### Large Molecular (Middle Molecule) Uremic Toxins and Uremic VC

Similarly, several studies have demonstrated that TNF-α and interferon-γ exposure induced VSMC calcification in cell models, and this process was reversible upon anti-inflammatory treatments [28]. These inflammatory cytokines are partially responsible for VC development through upregulating NF-κB and Wnt-3a/7a expressions, and activating early osteoblast differentiation signals such as Msx2 and osterix [29]. AGEs also exhibit a VC-inducing capacity through increasing the expressions of AGE receptors, elevating oxidative stress severity and triggering VSMC apoptosis [30]. These uremic toxins collectively account for the heightened risk of vasculopathy, especially VC, observed in patients with CKD and ESRD [2,12].

### 4.3. Protein-Bound Uremic Toxins 

These are among the most influential factors that contribute to adverse influences including several prototypes such as IS and *p*-cresyl sulfate (pCS) [31]. Dietary aromatic amino acids, after being deaminated and decarboxylated by colonic microbes, become phenolic compounds such as *p*-cresol, which is processed to pCS through detoxification in liver as well as colonic mucosa. Dietary tryptophan, on the other hand, is converted to indole by colonic microbes, which is further metabolized by liver into IS. Both IS and pCS act as typical uremic toxins by showing direct cytotoxic effects to hepatic cells, myocardium, and renal tubular cells, in animal experiments and human studies [32].

#### Protein-Bound Uremic Toxins and Uremic VC

Their crucial role in the pathogenesis of VC is increasingly recognized [33]. Aside from stimulating the activity of various NADPH oxidases (Nox) and suppressing anti-oxidant levels, IS causes higher oxidative stress and alters the proliferative ability/survival of cardiomyocytes and VSMCs, thus setting the background of subsequent VC appearance in uremic patients [34]. In the uremic environment, IS has also been found to induce CpG hypermethylation involving Klotho in VSMCs, silencing these instrumental calcification inhibitors and predisposing them to the development of VC [35]. Vasoactive miRNAs, including miR-29b [36] and miR-125b [21], have been shown to be suppressed, leading to Wnt-7b/β-catenin up-regulation in IS-triggered VC in experimental models. The VSMC fate of progressive phenotypic change to osteoblast-like cells upon IS exposures also involves PI_3_K/Akt pathway upregulation [37]. Specifically, high phosphate condition can upregulate PiT-1 expressions and increase calcium deposition in treated VSMCs [38]. In addition, IS activates the proinflammatory macrophages through notch signaling to accelerate atherogenesis and potentially VC [39]. It is also demonstrated that CKD rats undergoing long-term pCS feeding had greater aortic and peripheral arterial calcifications, accompanied by coagulation cascade activation and aggravated inflammation [40]. In light of findings from these reports, protein-bound uremic toxins, in serum levels approximating those in ESRD patients, appear to be a main driving force for perpetuating VC and aggravating its severity.

**Figure 1:**
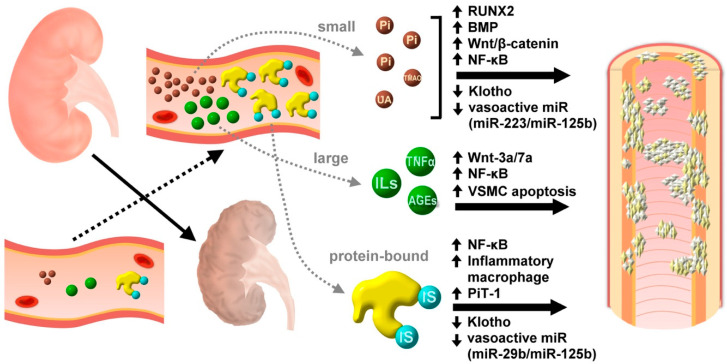
A schematic diagram showing the potential contribution of different size groups of uremic toxins, from small molecules, large molecules, to protein-bound ones, to the development of uremic vascular calcification. AGE, advanced glycation endproduct; BMP, bone morphogenetic protein; IL, interleukin; IS, indoxyl sulfate; miR, microRNA; Pi, inorganic phosphate; TMAO, trimethylamine-N-oxide; TNF, tumor necrosis factor; UA, uric acid; VSMC, vascular smooth muscle cell.

A summary of the pathogenic connection between uremic toxins and uremic VC is shown in Figure 1.

## 5. Existing Options of Therapeutic Uremic Toxin Reduction for Managing VC

**Figure 2:**
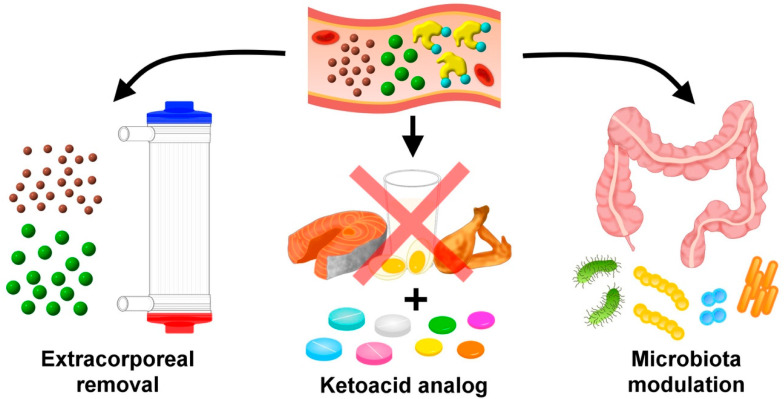
A brief summary of existing strategies for reducing uremic toxin levels and their related complications, especially uremic vascular calcification.

Judging from the importance of uremic toxins to cause uremic VC, it is tempting to devise treatments against VC by lowering these toxins (Figure 2). There has been a call for adopting similar strategy to manage other difficult-to-treat complications stemming from CKD [41]. Several approaches have been attempted, as simply outlined below.

### 5.1. Extracorporeal Toxin Removal

Artificial kidneys aiming to remove circulatory uremic toxins are the cornerstones for this toxin-directed approach. Conventional dialytic modalities efficiently extract small molecules like inorganic phosphate and uric acid (Figure 2). However, for larger molecular toxins such as interleukins, TNF-α, and FGF-23, alternative dialytic regimens are frequently required to enhance their removal. The reduction of protein-bound uremic toxins through the existing dialytic modalities is particularly difficult and inefficient. More complex material and biomechanical design are needed in order to overcome these barriers [42].

### 5.2. Dietary Modification with Ketoacid Supplementation

Dietary manipulation is an alternative approach to reduce uremic toxins (Figure 2). Several small-scale studies have shown that a very low protein diet combined with ketoacid analogues supplementation potentially reduces uremic toxin production among patients with advanced CKD [43]. Moderation of dietary animal proteins with an increasing fiber content was found to lower protein-bound uremic toxin generation in patients with an estimated glomerular filtration rate (eGFR) < 60 mL/min/1.73 m^2^ [44]. A substantial concern that long-term dietary approach for toxin reduction may introduce malnutrition and neutralize the original benefits remains. Several features of these dietary modifications are also challenging to patients’ perseverance [45].

### 5.3. Interventions through the Gastrointestinal Tract: An Emerging Approach

Treatments delivered through the gastrointestinal tract are now gaining attention in modern research to manage toxin-related complications. Indeed, the importance of a kidney-gut axis has been upheld in recent years. Progressive renal function impairment is frequently accompanied by a deranged gastrointestinal bacterial homeostasis (dysbiosis), owing to uremia-induced gastrointestinal motility and permeability changes via the direct effect of uremic toxins and/or their metabolites on colonic microenvironment [46]. Gut dysbiosis, in turn, contributes to adverse vascular remodeling (kidney–gut–vascular effects) and an increased cardiovascular risk among patients with ESRD [46]. The altered host gastrointestinal microenvironment increases the rate of colonic protein fermentation, leading to a greater production of IS and pCS, both experimental precipitators of uremic VC [46,47,48]. It seems to be promising to target the gastrointestinal tract, especially gut dysbiosis, as a means to ameliorate cardiovascular morbidity and even VC in renal patients [49].

## 6. Gastrointestinal Decontamination for Toxin Removal and VC Counteraction

**Figure 3:**
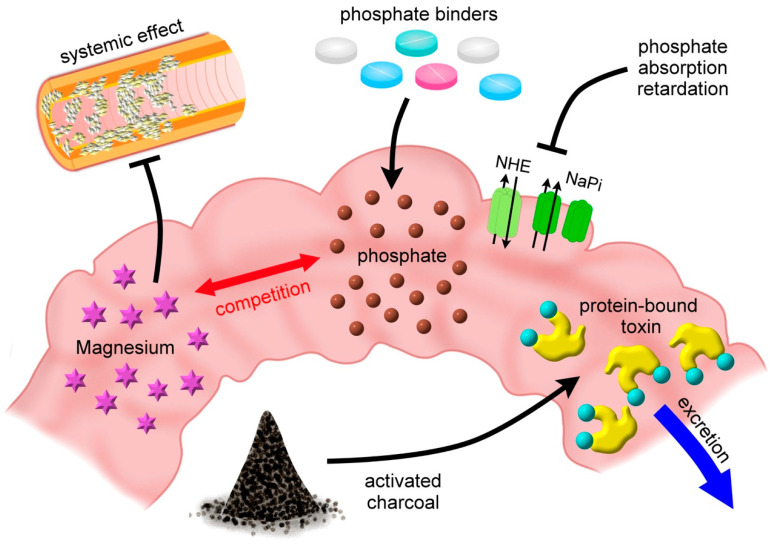
Promising approaches against vascular calcification based on gastrointestinal decontamination for uremic toxins. NHE, sodium-hydrogen exchanger; NaPi, sodium-phosphate cotransporter.

Apart from microbiota modulation, there are other plausible interventions aiming to reduce uremic toxins through the gastrointestinal route. The concept of “gastrointestinal decontamination” has been embraced by the healthcare community for treating poisoning for decades. Through the timely removal of harmful substances and the prevention of gastrointestinal toxin absorption, gastrointestinal decontamination assists in optimizing patient outcomes. Recently, this approach is further adopted from diverse fields, for example, the use of non-absorbable antibiotics to exert gastrointestinal decontamination on attenuating intestinal inflammation and reducing the risk of graft-versus-host disease in patients undergoing hematopoietic stem cell transplantation [50] and selective decontamination of the digestive tract (SDD) in critically ill patients with various infections (ventilator-associated pneumonia, and resistant bacteria colonization) [51,52]. Based on these relevant and positive findings, we propose and repurpose that “gastrointestinal decontamination” may be reasonably adapted for uremic toxin to ameliorate uremic VC, as detailed below (Figure 3).

### 6.1. Gastrointestinal Phosphate Unloading 

Tight control of the serum phosphate level reduces VC through the reduction of secondary hyperparathyroidism, calcium phosphate product, and active mineralization protein deposition in vascular cells [53]. In addition to the restriction of dietary organic and inorganic phosphate intake, several medications are also capable of removing phosphate or decreasing phosphate absorption from the gastrointestinal tract.

#### 6.1.1. Phosphate Binders

Each class of these phosphate binders has their strengths and drawbacks (Table 1) (part of the content from [53,54]). Traditional phosphate binders, especially calcium-based ones, fail to decrease VC extent, presumably because of the efficacy offset by their calcium burden. A recent cohort study showed that calcium-based phosphate finders significantly increased the risk of cardiovascular events compared with non-users among patients with pre-dialysis CKD [55]. Newer classes of phosphate binders, including lanthanum carbonate and sevelamer, have been anecdotally shown to retard coronary artery calcification and modestly lower pulse wave velocity in CKD and ESRD patients [56,57], as well as mortality in CKD patients [58]. However, their ability to slow the extent of VC seems lost in those with normal serum phosphate levels, casting doubt on the exact efficacy of these newer non-calcium phosphate binders [59]. Iron citrate, a more recent member of phosphate binders, ameliorates vascular calcification through decreasing gastrointestinal phosphate availability and absorption in animal models of CKD and potentially in clinical settings [60]. Oral iron citrate administration is also found to favorably modify extracellular matrices in vascular media constituents, reducing or even reversing calcification [61]. These data evidently support that the reduction of gastrointestinal phosphate quantity through selective binding can be a useful option for managing uremic VC. However, the use of non-calcium-based phosphate binders in those with earlier stages of CKD has not consistently altered the severity of CKD-mineral bone disorder (MBD) biochemically and anatomically. Furthermore, the types of phosphate binders have been shown to affect the bioavailability of intestinal vitamin K as an inhibitor of VC. An in vitro study hints at the possibility that calcium and lanthanum carbonate potentially bind to vitamin K, preventing their absorption [62]. Furthermore, the vitamin K-lowering effects of phosphate binders may also lead to impaired cholesterol metabolism [63], modifying the subsequent risk of atherosclerosis and VC as well.

#### 6.1.2. Inhibitors of Intestinal Phosphate Absorption

Inhibition of gastrointestinal phosphate absorption appears to be new plausible option for reducing the vascular toxicity posed by phosphate. Several small molecules including nicotinamide (a sodium/phosphate cotransporter inhibitor) and tenapanor (a minimally absorbed inhibitor of gut sodium/hydrogen exchanger) stand at the forefront of this approach [64]. Although nicotinamide use is shown to induce rapid and sustained reductions in serum phosphate levels among treated CKD patients [65], a recent study casted doubt on the presumed benefit of nicotinamide on VC. Kaesler et al. found that oral nicotinamide increased soft tissue calcification and VC in CKD mice as compared with gastrointestinal magnesium carbonate administration [66]. Tenapanor, acting via a non-phosphate binding mechanism, has been shown in animals to reduce intestinal paracellular and active transport of phosphate, increasing stool phosphorus content, while lowering urinary phosphate excretion [67]. Tenapanor was first tested in phase I to II trials for its hypophosphatemic efficacy, with favorable results generated [68]. A recent phase 3 randomized controlled trial demonstrated that tenapanor administered twice daily significantly reduced serum phosphate levels by 1.0 to 1.2 mg/dL over 8 weeks in hemodialysis patients [69]. This effect is also accompanied by a prominent reduction of FGF-23, suggesting that potential vascular benefits may exist despite unwanted side effects of diarrhea [68]. Tenapanor has been approved for use in patients with irritable bowel syndrome with predominant constipation in United States during 2019 [70], and is currently awaiting the approval of another indication, for serum phosphorus control in patients with dialysis-dependent ESRD. More evidence is currently needed to elucidate whether intestinal phosphate transport inhibition truly confers a vascular benefit in patients with CKD and ESRD.

### 6.2. Reduce Gastrointestinal Calcium Exposure 

It should be emphasized that uremia is a state of calcium overload [71]. Prior studies suggested that excess calcium load contributed to cardiac calcification in patients with ESRD [72]. Calcium load from the consumption of calcium-based phosphate binders apparently contributes to worsening VC through inducing unwanted hypercalcemia and compromised calcium dynamics. Clinically speaking, a meta-analysis involving randomized controlled trials comparing different phosphate binders indicated that calcium-based phosphate binders incur significantly more hypercalcemic episodes than sevelamer (vs. calcium, relative risk [RR] 0.3) or lanthanum carbonate (vs. calcium, RR 0.16) [53]. Hypercalcemia has been well iterated as a potential risk factor for VC occurrence and progression [5]. Experimentally, higher ambient calcium concentrations directly lead to medial aortic calcification potentially through up-regulating PiT-1 [73] and others. Furthermore, anecdotal reports revealed that dietary calcium amount could similarly augment the risk of coronary artery calcium in patients with CKD [74]. It would be prudent to limit the gastrointestinal exposure of calcium in order to attenuate subsequent risk of VC.

### 6.3. Magnesium Competition: A Value-Added Approach

Magnesium has gradually been recognized for its potent inhibitory effects on the calcification process involving vasculature. Epidemiological studies have consistently revealed that serum magnesium levels inversely correlate with the presence, the severity, and the rate of progression of VC in patients with CKD and ESRD [75]. In animals with CKD, the gastrointestinal administration of magnesium carbonate effectively controlled phosphate availability and lower serum phosphate levels [76]. Moreover, magnesium carbonate dose-dependently attenuated serum parathyroid hormone concentrations and lowered aortic calcium amount as compared with neutral results in the sevelamer group. The mechanisms through which gastrointestinal magnesium delivery ameliorate VC include the local competition for phosphate binding and, more instrumentally, the systemic effects of calcification inhibition besides the phosphate-lowering efficacy [75]. Magnesium is shown to interfere with the formation of calcium hydroxyapatite, inhibit VSMC L-type calcium channels, and alter the expression of calcium-sensing receptors in parathyroid glands [75].

Consequently, gastrointestinal toxin decontamination based on magnesium administration may theoretically bear both local and systemic benefits in terms of vasculo-protection.

### 6.4. Oral-Activated Charcoal Administration 

Oral charcoal-based removal of protein-bound uremic toxin may be a useful choice to counteract VC. In vitro studies already support the feasibility of this approach [77]. Researchers already harness oral adsorbents for managing uremic VC experimentally [78,79,80,81,82,83,84] and clinically [85,86,87]. The plausible mechanisms underlying this calcification-reduction effect of activated charcoal can be multi-faceted, including less reactive oxygen species production, attenuated vascular ageing [80], lowered inflammatory mediators, and fibrokines [82]. However, the dosage of oral-activated charcoal and the duration of treatment could be effect modifiers [80,82]. Clinically, there were three studies administering oral-activated charcoal as uremic toxin adsorbents to treat uremic VC in patients with non-dialysis CKD (Table 2). A randomized controlled trial involving patients with earlier CKD (3 to 4) found that 12 months of oral activated charcoal led to progressively lower coronary artery calcium scores in the treatment group than those using calcium carbonate [86]. However, Sakaguchi et al. revealed that this treatment did not slow down coronary artery calcification [87]. It may be more reasonable to selectively choose patients with more severe CKD and a greater degree of VC to address the utility of this approach.

### 6.5. Gut Microbiota Manipulation 

Preliminary studies revealed that microbiota modulation through manipulating dietary composition might attenuate the extent of atherogenesis in animal models [88]. The use of prebiotics consisting of non-digestible carbohydrates to pre-emptively change microbiota [89] has also been tested in patients with CKD and ESRD. Most of these clinical studies, albeit small-scale, yielded promising results, such as lower pCS generation rates with reduced serum IS levels in treated patients [90,91]. The probiotics harnessing live microorganisms to improve host health are similarly expected to exert a toxin-reduction effect. Indeed, the findings from pilot studies support the potential utility of probiotics in attenuating serum markers of inflammation and oxidative stress among patients with ESRD [92,93]. Nevertheless, the influence of prebiotics and probiotics on cardiovascular outcome or VC has not been tested. More vascular endpoints in design should be available in the near future.

## 7. Future Perspectives

The pathogenesis of uremic VC, from the current understanding, includes VSMC phenotypic switch upon noxious metabolic stimuli; rising oxidative stress; a pro-inflammatory environment; the declined anti-calcific factors; and, most importantly, the adverse influences posed by various uremic toxins. Small- and large-size uremic toxins as well as protein-bound ones each predispose CKD or ESRD patients to develop uremic VC. Interventions aimed to neutralize uremic toxins have since been embraced as potential therapeutic options for uremic VC. The gastrointestinal tract serves as both a source of uremic toxin generation and the route of treatment delivery. The concept of gastrointestinal decontamination through the elimination or the prevention of toxin absorption within the gut can be an integrative approach for ameliorating VC. Administration in combinations to target uremic toxins is expected to counteract VC from different dimensions and act synergistically. The quest for an optimal VC management strategy in CKD and ESRD is still underway [94]. Hopefully, we will be able to have a new choice in our armamentarium against uremic VC through the gaining of more understanding in this field.

## Figures and Tables

**Table 1:** Comparisons of features between different phosphate binders.

Phosphate Binders	Binding Efficacy *	Calcium Load *	Effect on FGF-23	Effect on PTH	Effect on 1,25-(OH)_2-_vit D	Effect on Survival	Side Effects
Calcium-based (carbonate, acetate, citrate)	+	+ ~ +++	None	Decrease	Decrease	None	Hypercalcemia, ectopic calcification
Non-calcium-based							
Magnesium-based	+	−	?	Decrease	Increase (potential)	None	Diarrhea, magnesium overload
Iron-based	+ ~ ++		Decrease	Decrease	Increase (potential)	None	Diarrhea, iron overload
Sevelamer	+ ~ ++	−	Decrease	Decrease	Increase	Improve	Constipation, metabolic acidosis (if HCl group)
Aluminum hydroxide	+++	−	?	Decrease	?	None	Aluminum toxicity, adynamic bone disease, constipation
Lanthanum carbonate	++++	−	Decrease	Decrease	None	Improve	Constipation

FGF-23, fibroblast growth factor-23; PTH, parathyroid hormone; vit D, vitamin D. * For describing the strength of influences (each column) associated with each phosphate binder, the symbols +, ++, +++, and ++++ are used in order of perceived increasing efficacies. The minus (−) symbol represents the absence of such influence.

**Table 2:** Clinical studies focusing on the therapeutic effect of uremic toxin adsorbents on VC in patients with CKD.

Adsorbent Types	Dose	VC Measurement Methods	Effects	Baseline Renal Function	Number of Patients	Reference
AST-120	5.1 ± 1.4 g/d	Abdominal aortic calcifications in abdominal CT	Lower aortic calcification index in users	Stage 4 to 5 (pre-dialysis)	199	[85]
Activated charcoal	1.8–3.6 g/d	Coronary artery calcifications in multidetector CT	Lower coronary calcium scores in users	Stage 3 to 4	97	[86]
AST-120	6 g/d	Coronary artery calcifications and thoracic aorta calcifications in multidetector CT	No differences in coronary calcium scores or aortic calcification	Stage 3 to 4	96	[87]

CKD, chronic kidney disease; CT, computed tomography; VC, vascular calcification.

## Abbreviations

AGEs, advanced glycation endproducts; BNP, bone morphogenetic protein; CKD, chronic kidney disease; CRIC, Chronic Renal Insufficiency Cohort; eGFR, estimated glomerular filtration rate; ESRD, end-stage renal disease; EUTox, European Uremic Toxin; FGF-23, fibroblast growth factor-23; IL-1β, interleukin-1β; IS, indoxyl sulfate; NF-κB, nuclear factor-κB; Nox, NADPH oxidases; pCS, *p*-cresyl sulfate; RR, relative risk; SDD, selective decontamination of the digestive tract; TMA, trimethylamine; TMAO, trimethylamine-N-oxide; TNF-α, tumor necrosis factor-α; VC, vascular calcification; VCAM, vascular cell adhesion molecule; VSMC, vascular smooth muscle cell.
